# Regulation of Hedgehog Signalling Inside and Outside the Cell

**DOI:** 10.3390/jdb4030023

**Published:** 2016-07-20

**Authors:** Simon A. Ramsbottom, Mary E. Pownall

**Affiliations:** 1 Institute of Genetic Medicine, International Centre for Life, Newcastle University, NE1 3BZ Newcastle upon Tyne, UK; 2 Biology Department, University of York, YO10 5YW York, UK; betsy.pownall@york.ac.uk

**Keywords:** signalling, regulation, heparan sulfate, Hedgehog

## Abstract

The hedgehog (Hh) signalling pathway is conserved throughout metazoans and plays an important regulatory role in both embryonic development and adult homeostasis. Many levels of regulation exist that control the release, reception, and interpretation of the hedgehog signal. The fatty nature of the Shh ligand means that it tends to associate tightly with the cell membrane, and yet it is known to act as a morphogen that diffuses to elicit pattern formation. Heparan sulfate proteoglycans (HSPGs) play a major role in the regulation of Hh distribution outside the cell. Inside the cell, the primary cilium provides an important hub for processing the Hh signal in vertebrates. This review will summarise the current understanding of how the Hh pathway is regulated from ligand production, release, and diffusion, through to signal reception and intracellular transduction.

## 1. Introduction

Hedgehog (*hh*) was originally identified as a segment polarity gene in *Drosophila* [1]. Its name comes from the mutant phenotype of the larvae, where the neatly patterned rows of denticles are disrupted and instead a lawn of denticles form, resembling the spines of a hedgehog. *Drosophila* only has one *hh* gene, whereas vertebrates have three (or more) and, of these, Sonic hedgehog (*Shh*) has garnered the greatest interest due to its beautiful and specific expression in major signalling centres of the vertebrate embryo [2,3,4]. Since the initial description of Shh, it has been found to be an essential regulator in a wide range of contexts during vertebrate development, and was the first morphogen proven to act in vertebrates, where it diffuses to pattern the different neural cell types in the ventral neural tube in a concentration-dependent manner [5].

The discoveries made while investigating the molecular and cellular basis of the hedgehog signalling pathway have revealed novel and unpredicted mechanisms. For instance, the recognition that primary cilia serve as a signalling hub was an unexpected finding from a forward genetic screen for embryonic patterning defects similar to the *Shh* mutant phenotype [6]. While this finding was made in mice, the complex machinery of kinases and microtubule-associated proteins involved in the intracellular relay of the hedgehog signal were largely dissected genetically in *Drosophila*; again, an unbiased genetic approach was essential for the discovery of many components of the Hh signal transduction pathway. Biochemical analyses [7,8,9] have also been crucial in discovering the extensive processing of the hedgehog ligand, revealing the fatty nature of the ligand that was difficult to reconcile with its long range morphogen functions in the *Drosophila* wing disc and the vertebrate neural tube and limb bud [10]. In addition to what is termed the “canonical” signalling pathway, Hh signalling can be elicited outside of this pathway to affect a number of diverse roles, including regulation of proliferation, chemotaxis, and cell motility using a “non-canonical” pathway that has been reviewed elsewhere [11,12]. This review will focus on the regulation of canonical hedgehog signalling, from expression of the ligand through to the activation of target genes.

## 2. Hedgehog Expression

Regulation of hedgehog gene expression was first identified in *Drosophila*, where the expression of *engrailed* (*en*) within the posterior compartment of the wing disc specifies posterior identity and drives *hh* expression in the same compartment [13]. Ectopic En protein in the anterior compartment results in ectopic *hh* activation, while loss of *en* in the posterior compartment results in a loss of *hh*. In the eye imaginal disc, however, *en* is not required for *hh* expression [14], suggesting a role for multiple regulatory factors. Additional transcriptional regulators have been identified including *Master of thickveins* (*mtv*), and *Serpent* (*Srp*), which act to restrict the expression of *hh* in anterior cells and activate *hh* during haematopoiesis, respectively [15,16]. In vertebrates, a screen identified Hepatocyte nuclear factor 3 (Hnf3)-class transcriptional regulators, which bind some but not all *Shh* enhancers [17], driving expression of *Shh* in the notochord and floorplate, where it acts to pattern the neural tube [18]. The homeobox transcription factor *Aristaless related* (ARX) similarly acts to promote *Shh* within the neural tube, working in conjunction with FOXA2 (previously known as HNF3β) within the ventral neural tube [19]. Ectopic expression of HNF3β (FoxA2) results in ectopic activation of *Shh* [20], and misexpression of proneural transcription factors. *Shh* is also expressed within the posterior margin of the developing tetrapod limb bud, in a region known as the Zone of Polarising activity (ZPA), where it acts to specify positional identity along the anterior–posterior axis. Expression of *Shh* within the ZPA is controlled by a cis-enhancer located a megabase upstream of *Shh* within intron five of the *Lmbr1* gene [21]. This ZPA regulatory sequence (ZRS) contains binding sites for the ETS transcription factors *Ets1*, *Ets2* *Etv4*, *Etv5*, *Elf1* and *Gabpα*, which act to delineate the expression boundary of *Shh* while simultaneously repressing ectopic expression of *Shh* outside the ZPA [22].

Hedgehog signalling is crucial for the development of a number of different embryonic systems, and consequently its expression is tightly regulated by the combined action of multiple transcription factors. Mutations within transcription factor binding elements, or of the transcription factors themselves, can therefore significantly affect hedgehog expression, resulting in a number of conditions associated with aberrant hedgehog signalling including polydactyly, tibial hypoplasia, X-linked lissencephaly, and epilepsy [21,22,23,24,25,26].

## 3. Ligand Synthesis

**Figure 1:**
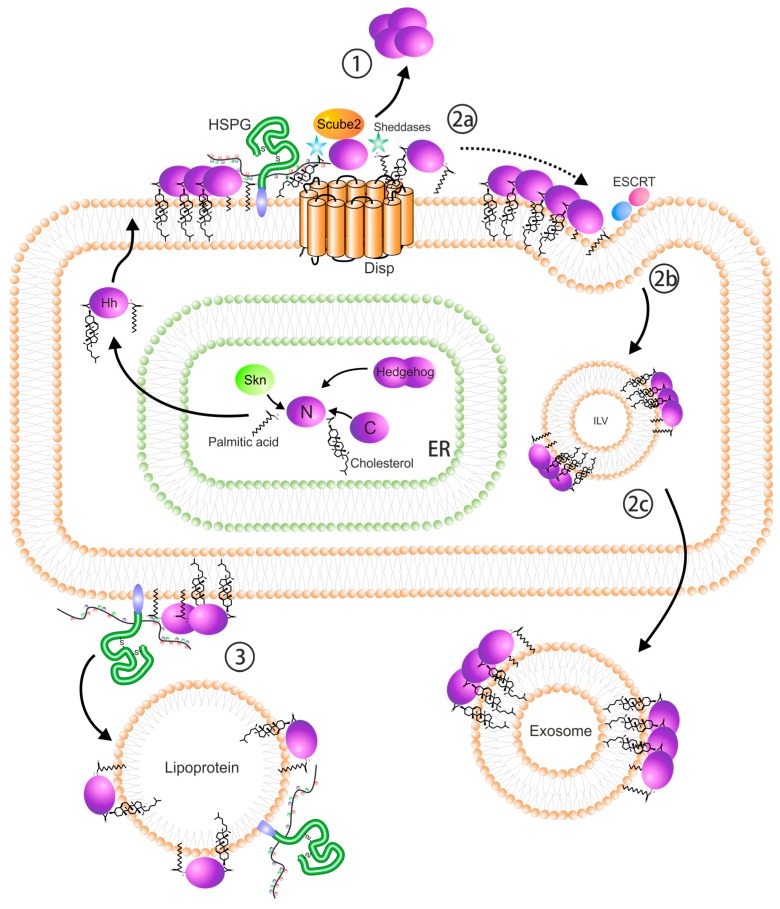
The sonic hedgehog (Shh) ligand is initially synthesised as a precursor with two distinct domains: the N-terminal “hedge” and C-terminal “hog” domain, which undergoes autoproteolysis to give N- and C-terminal fragments. The C-terminal fragment acts as a cholesterol transferase to attach cholesterol to the N-terminal fragment. Skinny hedgehog attaches palmitate to the N-terminus of hedgehog (Hh) to give rise to the fully processed form. Following synthesis, Hh is delivered from the endoplasmic reticulum to the cell membrane, where it is released from the cell via a number of different mechanisms. (**1**) Hh binds Disp in a cholesterol-dependent manner, and through the combined action of Disp and Scube2, is released from the cell. Heparan sulfate proteoglycans (HSPGs) act as assembly points for multiple components. Hh monomers are able to form multimeric complexes aided by association with HSPGs and Scube2. Hh is released following proteolytic processing by sheddases, which remove cholesterol and palmitate. These complexes are more soluble than the monomeric form and so are able to diffuse away from the cell; (**2**) Unprocessed Hh may re-enter the cell in a Disp-dependent fashion (**2a**) and be internalised by endosomal sorting complexes required for transport (ESCRT) proteins, which sort Hh proteins into intra-luminal vesicles (**2b**); These vesicles subsequently fuse with the plasma membrane and are released from the cell (**2c**); (**3**) Association of Hh with HSPGs results in loading of Hh into lipoprotein particles. Glypicans (Glycosylphosphatidylinositol (GPI)-linked HSPGs) may be cleaved and released along with Hh.

The hedgehog ligand is initially synthesised as a 46 kDa precursor [7,8], with two distinct domains: the N-terminal “hedge” domain is processed to a 19 kDa fragment (Hh-N) following proteolytic cleavage that is executed by the C-terminal “hog” domain [10] within the endoplasmic reticulum [27] (Figure 1). The C-terminus acts as a cholesterol transferase to covalently attach a cholesterol group to the carboxy end of the Hh amino terminal fragment, Hh-N [8,28]. The nascent Hh-N is further modified by the subsequent addition of a palmitoyl group at Cys-24 [9], resulting in an extremely hydrophobic molecule that is referred to as Hh-Np for Hh-N-processed. The processing of Hh-N takes place in the secretory pathway and is mediated by a palmitoylacyltransferase which is coded for by the Skinny hedgehog gene (*Ski/Skn*) [29]. This gene is also known as *sightless* (*sit*) [30], *rasp* [31], and hedgehog aceyltransferase (Hhat) [32]. Mice deficient in *Skn* exhibit similar defects to *Shh* mutants: they lack a differentiated floor plate and display neural tube defects. These data indicate that the palmitoyl addition is essential for SHH function [33]. The addition of cholesterol and palmitate increases the efficacy of SHH-Np [33,34], while addition of hydrophilic adducts to the N terminus reduces the activity of SHH [35]. Overall, the consensus is that lipidation of the Hh signalling protein is an essential step in producing a functional signalling molecule.

## 4. Ligand Release and Dispersal

The dual lipidation of Hedgehog results in a very hydrophobic molecule that should find it difficult to move very far from the cell membrane. This is evident in tissue culture experiments: when the lipid-modified form of SHH (SHH-Np) is expressed in cells, it is found associated with cells, and is not released into the surrounding medium [7,36,37]. In contrast, when a form of Hedgehog that has no cholesterol adduct (termed Hh-Nu) is expressed, it is released by cells in the media [37]. Hh-Nu has also been shown to have an increased range compared with Hh-Np [38], further supporting the idea the cholesterol has a role in Hedgehog tethering. These experiments are important to note because they are in sharp contrast to the well-documented ability of SHH to act as a morphogen in vivo. A morphogen is a signalling molecule that can elicit different responses depending on its concentration. Therefore, a morphogen is present as a gradient. Consistent with this, SHH has been shown to display the characteristics of a freely-diffusible molecule, and is able to act from a distance in a morphogenic manner [39]. Due to the extensive lipid modification of the Hh ligand, the a priori assumption would be that Hh should be tethered to the membrane, thus precluding its ability to diffuse and act as a morphogen. The ability of Hh to signal at a distance, however, suggests that modification of the protein is not for the purpose of protein restriction but instead forms part of a regulated process, tightly controlling the release and subsequent diffusion of Hh ligands.

Evidence for the regulated release of Hh ligands was found when the membrane protein *Dispatched* (*Disp*) was shown to be required for the release of Hh-Np [40]. Disp contains a sterol sensing domain (SSD), which can be found in other proteins that directly bind cholesterol [41]; Disp binds SHH in a cholesterol-dependent manner [42], and loss of Disp leads to retention of Hh-Np in producing cells [40]. Hh-Nu, however (which is not cholesterol modified), is not retained following loss of Disp, suggesting that addition of cholesterol during ligand processing is responsible for Hh tethering at the membrane, and that this is overcome by the action of Disp [40,42]. Mice homozygous null for *Disp* show the characteristic phenotype resulting from a lack of hedgehog signalling; they exhibit cyclopia and holoprosencephaly and display reduced expression of *Shh* target genes [43]. The failure of SHH and Hh to be released from its site of synthesis in the absence of Disp in both flies and mice indicates a conserved mechanism for Hedgehog release. DISP alone, however, is not sufficient for the release of cholesterol-modified SHH from cells, but instead requires the action of a secreted protein—SCUBE2. The interaction of SCUBE2 is also dependent on cholesterol, and the differential affinity of SCUBE2 and DISP for different regions of the cholesterol molecule, suggest a hand-over mechanism between the two proteins, which promotes SHH release from the membrane [42]. SCUBE2 also forms an interaction with sheddases [44] which, with the support of heparan sulfate proteoglycans (HSPGs) [45], trim off the lipid groups with which hedgehog proteins interact with the plasma membrane, and so promote Hh release into the extracellular space (Figure 1-1).

**Figure 2:**
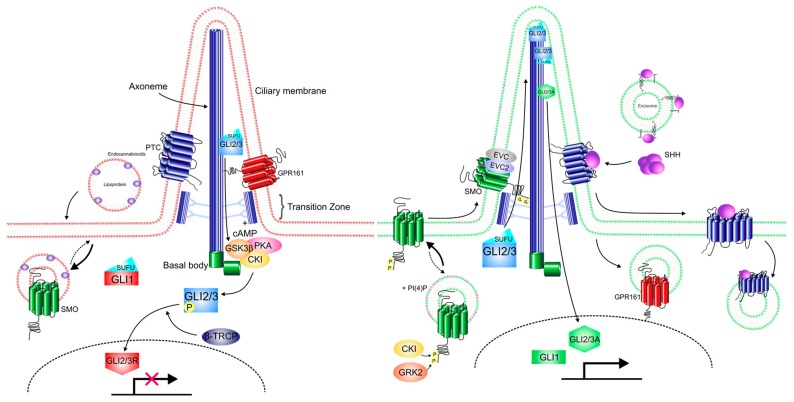
(**Left**) In the absence of hedgehog ligand, patched (PTC)—which is enriched in the cilial membrane—acts to repress smoothened (SMO) through the recruitment of lipoproteins and regulation of their phospholipid composition. SMO is maintained in its inactive state and sequestered within the cell. Upon exiting the cilium, GLI2 and GLI3 are phosphorylated by glycogen synthase kinsase 3β (GSK3β), casein kinase I (CKI), and protein kinase A (PKA); the activity of PKA is promoted by elevated cAMP levels due to the presence of G-protein-coupled receptor 161 GPR161. Phosphorylated GLI is recognised by β-TRCP, which promotes ubiquitylation and degradation of its C-terminal domain, giving rise to a cleaved repressor form. This cleaved repressor translocates to the nucleus and represses hedgehog target genes. GLI1 is not cleaved but is sequestered within the cytoplasm by suppressor of fused (SUFU), preventing it from activating downstream signalling; (**Right**) Upon binding of hedgehog, the hedgehog-PTC complex is internalised, and SMO inhibition by PTC is released; endocannabinoid levels are reduced, while phosphatidylinositol-4 phosphate (PI(4)P) levels increase, promoting SMO accumulation at the membrane. SMO is phosphorylated by CK1 and GRK2, leading to its activation. Activated SMO accumulates within the cilial membrane and binds EVC and EVC2. GPR161 exits the cilium and is internalised. GLI proteins within the cilial tip dissociate from SUFU and translocate the nucleus to activate Shh target genes.

SHH can be released from cells in multimeric complexes, the formation of which depends on N-terminal palmitoylation and the addition of cholesterol [33,34,46,47]. HSPGs present at the cell surface provide a scaffold to allow the formation of Hedgehog multimers, presenting a platform to promote the accumulation of SHH on producing cells, thereby facilitating SHH oligomerization and release from the plasma membrane [47]. The multimeric complex of SHH is more stable [47] and more active [33] than its monomeric form. A form of SHH lacking palmitate, SHH-N, is unable to form multimeric complexes while retaining the ability to activate downstream signalling, indicating that this modification is an important level of regulation. When expressed in the developing limb bud, unmodified SHH-N is unable to signal effectively over any significant distance, underlining the observation that long-range signalling requires lipid modifications that promote the formation of multimers [48]. When presented at the cell surface, multimeric Hh complexes may be internalised by ESCRT (endosomal sorting complexes required for transport) proteins [49], and packaged into intraluminal vesicles (ILVs), which subsequently fuse with the plasma membrane and are released as exovesicles [50]; evidence in *Drosophila* suggests that this is a Disp-dependent process [51] (Figure 1-2). More evidence for multimeric Hh complexes come from the identification of exovesicles containing Hedgehog proteins in *C. Elegans*, *D. Melanogaster* and *Mus musculus*, suggesting this is an evolutionarily-conserved release mechanism [50,52,53]. Release of multimeric hedgehog into the extracellular space requires the action of DISP, SCUBE2, and sheddases [54]. The route of export may therefore be determined by the availability of sheddases at the cell surface; if sheddases are present, hedgehog is released in a multimeric lipid-free form into the extracellular space (Figure 1-1), whereas if they are not, Hh is internalised and exported via exosomes (Figure 1-2).

## 5. Heparan Sulfate Proteoglycans

Heparan sulfate proteoglycans (HSPGs) are large glycoproteins that are ubiquitously expressed and can be found both at the cell surface and within the extracellular matrix [55,56]. HSPGs are comprised of a core protein covalently linked to long, unbranched glycosaminoglycan (GAG) chains [57]. The heparan sulfate (HS) chains can be modified by sulfation during their synthesis in the golgi, giving rise to a high degree of heterogeneity along their length. The first evidence demonstrating an essential role for HSPGs in the regulation of hedgehog signalling came from *Drosophila* mutants. Mutation of any of the genes coding for the enzymes required for HS synthesis (the UDP glucose dehydrogenase *sugarless*, the N-deacetylase/N-sulfotransferase *sulfateless*, the glycosyltransferases *tout-velu*, *brother of tout-velu*, *sister of tout-velu*), or the proteoglycans themselves (the glypicans *dally* and *dally-like protein*) results in disrupted hedgehog signalling [58]. Glypicans, Glycosylphosphatidylinositol (GPI)-linked HSPGs, are able to bind the lipoprotein lipophorin, and its recruitment to Hh signalling cells promotes the loading of Hh into soluble lipoproteins [59] (Figure 1-3). Furthermore, this association of Hh with lipophorin has been shown to be required for the full signalling activity of Hh [60]. Vyas, et al. (2008) visualised nanoscale oligomers of Hh that are associated with HSPGS at the surface of ligand producing cells [61]. The relevance of HSPGs in hedgehog signalling was brought into question however when a mouse carrying a mutation in the Cardin–Weintraub (CW) motif of *Shh*, a domain important for the ligand interaction with HSPGs, showed decreased SHH-HSPG interaction, but displayed no patterning defects associated with *Shh* disruption [62]. These data suggest that the interaction of the CW domain of SHH with HSPGs is not important for its ability to pattern the neural tube. However, a study [63] examining the crystal structure of SHH complexed with heparin found that a SHH dimer forms a continuous stretch of positive amino acids that interact with the heparin chain, and concluded that both the CW motif and the newly described “core GAG-binding site” contribute to SHH interactions with HSPGs.

Some evidence suggests that HSPGs can act as co-receptors for Hh signal transduction; a role for HS as a co-receptor is already well-established for other signalling pathways, such as Fibroblast growth factor (FGF) signalling (reviewed by [64]). The important role for glypicans in hedgehog signalling in *Drosophila* and vertebrates has been recently reviewed [65]; in short, glypicans can act to either sequester Hh ligand to prevent signalling or, in other cases, glypicans can stabilise the association of the ligand with the Ptc receptor to promote signalling. Opposite effects of glypicans in modulating hedgehog signalling have been described in mice and *Drosophila*, where GPC3 acts as a negative regulator of SHH activity by competing with PTC1 for SHH binding, while dally-like protein (Dlp) allows Hh to interact with Ptc1 [66,67]. These differences could be species- or context-dependent; alternatively, it is possible that different structural modification of the HS chain could underlie these contrasting findings. Recently, GPC5 carrying a specific sulfation modification (2OS-Iro) to the GAG chain has also been identified as a *Shh* co-receptor, as it is enriched in cells responsive to SHH and can promote downstream signalling [68].

## 6. Regulation by SULF1

Structural modification of heparan sulfate chains by the cell surface endosulfatase SULF1 plays a significant role in regulating the establishment of a gradient of Hedgehog ligand during the development of the *Drosophila* imaginal disc [69] and the vertebrate neural tube [70,71,72]. SULF1 activity hydrolyses a sulfate group from the 6O position of glucosamine sugars in highly sulfated HS chains [73]. The notable expression of *Sulf1* in the floorplate points to a possible role in *Shh* signalling, and Cathy Soula’s lab has used several vertebrate models to demonstrate that *Sulf1* expression is essential to the enhancement of *Shh* signalling in a cell-autonomous manner within the ventral spinal cord, and that this happens in precursor cells that require an increase in *Shh* signalling at a specific time in order to form oligodendrocytes [69,70,71]. *Sulf1^−/−^* mice had fewer cells expressing the oligodendrocyte transcription factor *Olig2* in the mantle zone compared to wild type, due to a failure of motor neuron precursors to make the switch to the oligodendrocyte lineage in the absence of *Sulf1*. These authors suggest that SULF1 acts to promote the production of active forms of SHH at the right time and place to induce specific neuronal cell types—an idea that is supported by the observation that HSPGs can promote the processing (removal of lipid groups) of active SHH at the cell surface [45].

A role for Sulf1 in modulating the diffusion of Hh in the *Drosophila* imaginal disc was demonstrated by Wojcinski, et al. [74], while prior to this, it was shown that Hh is unable to cross a field of cells lacking HSPGs [75,76]. These data suggest that HSPGs are essential for Hh diffusion, and that the nature of the HS structure is relevant for regulating this diffusion. Further evidence to support this notion came from studies in *Xenopus* explants, where the expression of *sulf1* leads to the retention of fluorescently-tagged SHH close to the cell membrane [72]. This same study showed that in the developing neural tube, the absence of *sulf1* resulted in a loss of *foxA2*-positive floorplate cells, which require a high level of SHH. Instead, *nkx2.2*-positive cells—which require lower concentrations of SHH—were found in the most ventral position. Further dorsally within the neural tube of *Xenopus* embryos, the *nkx6.1*-positive domain is extended, suggesting flattening and dorsal extension of the SHH gradient in the absence of *sulf1*. Consistent with this, direct visualisation of the endogenous ligand reveals an altered distribution, with ventral levels reduced and dorsal levels increased. These experiments demonstrate that SULF1 can limit the distance over which the Shh ligand can move, and thus increases the local concentration of the signal [72].

Further evidence of a central role for HSPGs in the recruitment of factors that facilitate release of the Shh ligand has recently been demonstrated [54]. The model supported by this data is that glypicans act as centres for the assembly of proteins that include SHH and adaptors such as SCUBE2, as well as proteolytic enzymes like sheddases; these cell-surface hubs promote the proteolytic release of SHH (Figure 1-1). The notion that proteolytic cleavage of the fatty side chains acts to promote Hh signalling seems at odds with previous research, as the palmitate group has been shown to be essential for Hh signalling [29] and long-range activity [33,47]. However, there is evidence that the proteolytic processing that releases the ligand is dependent on the N-palmitate [44,77], which anchors the protein while it is processed. An important observation from this same work [54] was that different cell types (which will be decorated with distinct HS chains with different levels of sulfation) vary in respect to how well they support the GPC/SCUBE2-dependent release of SHH. This is interesting, considering the reported differences in the release and diffusion of SHH from cells depending on whether they express the HS-editing enzyme Sulf1 [69,70,71,72]. It may be that SULF1 acts to influence the formation of these extracellular signalosomes to affect the release/distribution of Shh ligand.

HSPGs, and by extension, genes which act to modify HSPG structure, have a significant role to play in the release, dispersal, and reception of Hh. Manipulation of HSPGs can result in ligand trapping at the cell surface of cells producing the signal, or rapid diffusion away from the signalling cell. The gradient of ligand across a field of cells can be regulated, while reception of the signal at a receiving cell can be modified. In this way, HSPGs can be utilized to precisely define the range and level of activity of hedgehog signalling.

## 7. Regulation at the Membrane

The main receptor for the Hh ligand is the 12-pass membrane protein Patched (Ptc) [78]. In vertebrates, there are two patched genes, *Patched1 (Ptc1)* and *Patched2 (Ptc2)*, which are differentially expressed during development [79]. *Ptc1* null mice do not survive past embryogenesis, while *Ptc2* mutants are viable; *Ptc2* mutants develop alopecia and epidermal hypoplasia, however, indicating that neither protein can fully compensate for the loss of the other [80]. As well as the main receptor(s), several co-receptors for Hh signalling have been identified: Cell adhesion-associated, oncogene regulated (*Cdo)*/*interference hedgehog* (*ihog*) and Brother of Cdo (*Boc)*/*brother of ihog* (*boi*) are conserved between vertebrates and *Drosophila*, while a third, Growth arrest-specific 1(*Gas1*) is found only in vertebrates. Loss of *Cdo*/*ihog* or *Boc*/*boi* alone leads to minor changes in signalling, while inactivation of both results in loss of hedgehog signalling similar to that seen in the absence of Smoothened (Smo) [81,82]. In vertebrates, the membrane proteins CDO, BOC, and GAS1 interact with SHH and positively regulate signalling [82], while the co-receptor HHIP binds to SHH to negatively regulate signalling [83]. Recent evidence suggests that HHIP is not membrane associated, but rather a diffusible, extracellular regulator of Shh signalling, analogous to the well-documented bone morphogenetic protein BMP regulators like Chordin (reviewed by [84]). In the study that showed Hhip1 to have non-cell-autonomous effects in antagonising Hh signalling, it was interesting to note that the secretion of Hhip1 depended on its HS-binding motif, suggesting that its activity is modulated by HSPGs [85,86]—which, as discussed above, can also act as co-receptors for Hh signalling. A nice example of how hedgehog co-receptors can modulate SHH activity occurs during the development of the chick CNS, where SHH acts first as a chemoattractant and then as a repulsive cue as commissural axons descend to cross the ventral midline of the neural tube before they turn to continue their migration in an anterior direction. This navigation requires the expression of the glypican *Gpc1* in the commissural neurons, which, acting with SHH, promotes the expression of *Hhip1* to mediate a repulsive effect, directing an anterior migration of postcommissural axons [87,88].

Hedgehog signalling is unusual in its mode of activating downstream signalling; this is because its membrane-associated receptor Patched (Ptc) is an inhibitor of the pathway; so, rather than activating the Ptc receptor, the binding of the Hh ligand represses it. In the absence of ligand, Ptc acts to suppress the activity of a G-protein coupled receptor, Smoothened (Smo) [89]. The presence of ligand relieves the inhibition of the pathway by blocking the function of Ptc, allowing downstream signal transduction to occur. It is interesting, therefore, to observe that the depletion of the Hh receptor Ptc leads to ectopic activation of the pathway, while the lack of Smo results in a total loss of hedgehog signalling [89,90,91,92].

The inhibition of Ptc in response to ligand binding is generally agreed to be as a result of internalisation and degradation of the Ptc-Hh complex [93], however, the mechanism by which Ptc acts to inhibit Smo has yet to be fully elucidated. Originally, it was hypothesised that Ptc inhibited Smo via a direct physical interaction; Ptch and Smo display a high degree of co-localization in the absence of ligand [93,94], which is significantly reduced in Shh-responding cells [93,95]. Smo and Ptc are internalized together, but subsequently segregated within the endocytic pathway, allowing Smo to be recycled to a Ptc-negative compartment [94]. Ptc, however, acts in a substoichiometric way, able to repress signalling by half even when Smo is present in a 50-fold molar excess, suggesting a catalytic nature in its repressive activity [95], rather than direct physical inhibition. Ptc may therefore act by regulating the availability and/or activity of a Smo inhibitor or activator. Smo is known to be influenced by small molecules; for instance, the steroidal alkaloid cyclopamine is widely used to block Smo activity [96], and other drugs with this activity have been identified by screening chemical libraries [97]. The druggability of Smo, together with the structural similarity of Ptc to members of the RND (Resistance-Nodulation-Division) family of transporters, has led to the proposal that Ptc acts by importing or exporting small molecules to regulate Smo activity [98]. While the endogenous regulator of Smo is not known, a study by Bijlsma, et al. [99] showed that *Ptc*1-transfected cells display elevated levels of 3b-hydroxysteroid (pro-vitamin D3), which is released into the surrounding medium and can effectively block downstream signalling. Treatment of zebrafish with vitamin D3 leads to a loss of the *Shh* target gene *engrailed* expression in muscle pioneer cells and a down regulation of *ptc* expression, indicating reduced hedgehog signalling [99]. While vitamin D3 represses Smo, conversely, oxysterols (which are also an intermediate product in the cholesterol biosynthetic pathway) are able to activate Smo by binding a distinct region of its extracellular domain [100], resulting in the activation of Hh target genes [101]. Mutations in Smo which lead to the abolition of oxysterol binding reduce the level to which cells can respond to the Hh ligand, suggesting that these molecules may be required for endogenous signalling [100].

Other candidates for regulators of Smo activity are lipid kinases and phosphatases, given the importance of phospholipids in endosomal trafficking. Work in *Drosophila* has suggested a role for Ptc in the recruitment of lipoproteins to endosomes through which Smo is trafficked, resulting in the destabilization of Smo at the membrane [102]. Furthermore, a loss of Ptc activity leads to increased levels of the phospholipid phosphatidylinositol-4 phosphate (PI(4)P), which has been shown to promote Smo membrane localisation and activity [103], suggesting Ptc might act to control the nature of lipids in the membrane to affect Smo activity. Together, these studies point to a mechanism whereby Ptc acts to control the recruitment of lipoproteins and regulate their phospholipid composition. Recently, Khaliullina, et al. [104] undertook a screen for novel regulators of *Smo* by fractionating extracts of human very low density lipoproteins and testing the eluted fractions for the ability to repress activity of a *Shh* reporter line. A fraction showing inhibitory activity was analysed using lipid mass spectrometry and identified endocannabinoids as a class of lipids that inhibit Shh signalling, most likely at the level of SMO. Endocannabinoids are imported by lipoproteins; thus, if PTC acts to controls the level of PI(4)P in the membrane and the uptake of endocannabinoids, this would simultaneously prevent the accumulation and activity of SMO. Conversely, by blocking PTC activity, Hh promotes an increase in PI(4)P levels and a decrease in endocannabinoids, leading to the accumulation and activation of SMO. Interestingly, the finding that lipid-derived endocannabinoids regulate SMO activity means that there are likely to be additional mechanisms regulating *Shh* signal transduction through the activation or inhibition of endocannabinoid metabolism [104].

Smo is activated by phosphorylation at several sites in its C-terminal cytoplasmic tail [105]; this is thought to counteract electrostatic interactions that keep Smo in an inactive conformation [106]. Kinases involved in the hyperphosphorylation of Smo include casein kinase I (Ck1), G-protein-coupled receptor kinases (GRKs), and Protein Kinase A (Pka). However, while Smo is generally conserved, the sites phosphorylated by Pka in *Drosophila* are absent in mammalian SMO; it is, however, phosphorylated by CK1 and GRK2 [107]. In flies, Smo is initially phosphorylated by Pka [105,108,109], which promotes even tighter binding of Smo by Pka, leading to the formation of stable kinase-substrate complexes [110]. Smo is subsequently phosphorylated by CkI, CkII, and Gprk2 [105,109,111,112]. The sites on Smo targeted by GRKs are conserved throughout bilateria, suggesting that Smo regulation is fundamentally a GRK-mediated activity and GRKs are essential for activating downstream signalling [113]. However, recent work in zebrafish has indicated that while Grk2 functions as a positive regulator of Hh signalling, the genetic interactions do not support a model where Grk2 directly phosphorylates Smo, but instead regulates a step further downstream in the Shh pathway [114].

Phosphorylation of Smo by these kinases results in a conformational change in its structure. Smo contains multiple Arg clusters in its cytoplasmic tail, which together form an auto-inhibitory domain [106]. In the presence of ligand, phosphorylation of Ser/Thr residues results in the disruption of this inhibitory conformation, promoting Smo dimerisation and translocation to the cell surface. Smo activation appears to be a non-binary process, instead displaying differential levels of membrane accumulation and activation, depending on its level of phosphorylation [105,109]—a characteristic which may result from having numerous Arg clusters that are systematically disrupted by increasing levels of phosphorylation [106]. The more extensive phosphorylation of Smo promotes the expression of target genes that require high levels of Hh signalling [109]. Recently another regulatory component of this SMO activation complex has been identified. The Ser/Thr kinase CK1γ, known as *gilgamesh* (*gish*) in *Drosophila*, associates with SMO in response to Hh signalling, and phosphorylates its C-terminal tail to promote high-level pathway activation. Loss of CK1γ results in only subtle changes to Hh signalling levels, reinforcing the model of stepwise activation of SMO by kinases at multiple residues to precisely translate the concentration and longevity of the Hh ligand into a specific level of pathway activation [115].

## 8. Downstream of Smo

Activation of Smo leads to the up regulation of hedgehog target genes, a process mediated in *Drosophila* by cubitus interruptus (Ci). In the absence of Hh, Ci undergoes proteolytic cleavage [116] to form a truncated protein (Ci75), which acts as a transcriptional repressor [117]. In the presence of Hh, however, the full length Ci (Ci155) remains uncleaved and is able to activate Hh-responsive genes. Ci processing is regulated by a number of different proteins which act to sequester and phosphorylate Ci, controlling its activity and translocation to the nucleus [118]. A key regulator of Ci processing is the kinesin-related protein Costal-2 (Cos-2). Over-expression of Cos-2 promotes Ci cleavage and is sufficient to inhibit hedgehog signal transduction [119]. Conversely, loss of Cos-2 leads to Ci(155) accumulation, although this in itself is not sufficient to activate hedgehog signalling [119,120]. Cos-2 associates with both Ci [121] and Smo [122], as well as microtubules [123], and the affinity of Cos-2 for microtubules appears to be controlled by hedgehog, whereby its addition results in the release of Cos-2 [124]. By binding microtubules, Cos-2 provides the scaffold for a complex of proteins which contribute to the processing of Ci. This complex is comprised of four kinases: Protein Kinase A (Pka), casein kinase I (CkI), glycogen synthase kinase-3β (Gsk3β), the serine/threonine kinase Fused (Fu), as well as Ci [125]. Ci contains several Pka sites, suggesting that its cleavage may be facilitated by Pka phosphorylation, and mutation of these sites has been shown to be sufficient to inhibit Ci cleavage [126]. Similarly, loss of Pka function leads to accumulation of full-length Ci and subsequent activation of hedgehog target genes in the absence of Hh ligand [127]. Pka activity, however, does not seem to be moderated by hedgehog activity [128], suggesting that Pka activity is only a permissive factor in Ci regulation. Once phosphorylated, Ci binds to a component of the SCF (Skp, Cullin, F-box containing) ubiquitin E3 ligase complex—Supernumerary limbs (Slmb)—which acts to facilitate the processing of Ci into its repressor form [129], which then translocates to the nucleus to inhibit the transcription of hedgehog target genes.

Ci is also regulated by Suppressor of fused (Sufu). Sufu acts to sequester Ci within the cytoplasm in both its cleaved and full-length form, preventing its translocation to the nucleus [130,131]. In addition, Sufu is able to inhibit the transcriptional activity of Ci within the nucleus [131]. Therefore, in the absence of ligand, hedgehog signalling is repressed—both actively by the production of a repressor form of Ci and inhibition of transcriptional activity, and passively by the retention of unprocessed Ci within the cytoplasm. Binding of the Hedgehog ligand to Ptc leads to the release of Smo inhibition. Smo is phosphorylated by Pka, CkI, CkII, and Gprk2, and translocates to the membrane [105,108,111,112]. CkI, Pka, and Gsk3β dissociate, and the remaining parts of the signalling complex separate from microtubules [124]. Cos-2, Smo, and Fu form a complex that allows Fu to undergo autophosphorylation [132]. Subsequent CkI-dependent phosphorylation leads to full activation of Fu, which is then able promote Ci-155 stabilisation via the phosphorylation of Cos-2 and, along with CkI, inhibit the action of Sufu [132]. Activated Ci(155) translocates to the nucleus and interacts with CREB-binding protein (CBP) to activate target gene transcription [133].

In vertebrates, the Ci-related proteins GLI1, 2, and 3 [134] act as the effectors of the pathway and are differentially regulated by Shh signalling. GLI2 and GLI3 contain activator and repressor domains and can be proteolytically cleaved in a manner similar to that of Ci [135,136,137,138]. Phosphorylation of GLI2 and GLI3 promotes recognition by the F-box protein β-TRCP (homologue of Slmb), which targets them for processing [138,139]. Processing of GLI2, however, is very inefficient, and the small amount that is processed is rapidly degraded, resulting in very little cleaved GLI2 being present within the cell [135,139]. Conversely, GLI3 is efficiently processed into its repressor form [140]. This regulatory disparity is due to the fact that GLI2 and GLI3 contain a domain which confers differential susceptibility to proteolytic processing, which results in GLI3 being more efficiently processed than GLI2 [141]. Consequently, GLI2 functions primarily as an activator, while GLI3 acts predominantly in a repressive fashion. GLI1 does not contain the same processing domains as GLI2 and GLI3 and therefore acts exclusively as an activator [136,142]. Instead of undergoing cleavage to form a repressor, GLI1 is regulated by sequestration. In the absence of ligand, GLI1 is prevented from entering the nucleus by SUFU, which binds GLI1 and retains it in the cytoplasm; SUFU is also able to bind GLI within the nucleus and prevent it from activating target genes [143,144,145]. Upon signal activation, SUFU-GLI complexes dissociate, allowing GLI1 to enter the nucleus and activate signal transduction [146]. GLI1 can undergo alternative splicing to give rise to two variants, which either contain a truncation or a deletion within the N-terminal domain, termed GLI1ΔN and tGLI1, respectively [147,148], although only GLI1ΔN can be detected in normal (non-cancerous) tissue [148,149]. *Gli1* is transcriptionally up-regulated in response to *Shh* signalling [134,136,150], as are both of the alternative splice-forms of *Gli1* [147,148]. The presence of tGLI1 exclusively in cancerous tissue suggests that it may play a role in the transition from a healthy to a diseased state, an example of which can be observed in breast cancer, where tGLI1 is able to promote proliferation to a greater extent than GLI1 [149]. A full understanding of the expression and regulation of these isoforms is therefore crucial for the development of effective therapeutics [151].

A further difference in signal regulation between *Drosophila* and vertebrates is the respective importance of Fu and Sufu. While loss of Fu in *Drosophila* prevents downstream activation of Hh targets, there appears to be little effect in hedgehog signalling when a *fused* ortholog is knocked out in mice [152]. Alternatively, while Sufu in *Drosophila* is not a requisite part of the hedgehog pathway [153], its loss in mice leads to embryonic lethality stemming from constitutive activation of hedgehog signalling [154,155]. Another mechanistic difference between *Drosophila* and vertebrate Hh signalling can be observed at the level of the Smo–Cos-2 interaction. Requisite Cos-2 binding domains located within dSmo (*Drosophila*) are not necessary for mSmo (vertebrate) function; replacement of the C-terminal domain of mSmo with dSmo, however, renders the same Cos-2 binding sites in mSmo essential [156].

## 9. The Primary Cilium

While many of the mechanisms regulating hedgehog signalling are remarkably similar between vertebrates and *Drosophila*, one fundamental difference is the requirement of the primary cilium (PC) in vertebrates [6,157]. The primary cilium is an organelle that protrudes from the cell and has both a sensory and a signalling role [158]. It is comprised of a basal body formed from the mother and daughter centrioles, a central bundle of microtubules known as the axoneme, a complex of proteins at the proximal end known as the transition zone or “ciliary gate”, and the ciliary membrane (Figure 2). While the ciliary membrane is essentially a continuation of the cell membrane, transport in and out of the cilial space is highly regulated [159]. The transition zone contains multiple subsets of protein complexes, each named for the diseases which arise following their disruption. These include the nephronophthisis (NPHP) module, the Joubert Syndrome (JBTS) module, and the Meckel syndrome (MKS) module [158]. While disruption of these complexes can lead to specific syndromes, mutations of some key proteins (such as Cep290) can give rise to a range of different diseases [160].

Murine models of these mutations have suggested that in addition to site-specific mutations, the severity of the phenotype may be modified by additional genetic interactions [161]. The PC acts as the hub for Hh signalling activity; receptors, repressors, and downstream activators are in continuous flux, transiting through the ciliary space in a dynamic fashion [146,162,163,164]. Mutations of proteins which either regulate intraflagellar transport—which is required for movement through the cilial compartment—or of the transition zone—which controls entry into the cilium—give rise to defects in hedgehog signalling [6,157,159,165].

The proteins involved in GLI processing—PKA, CKI and GSK3β—are all localised to the basal body of the primary cilia [166,167,168], where they act to maintain the pathway in an inactive state [169] (Figure 2). The Ser/Thr kinase CK1γ—which phosphorylates SMO to promote pathway activation—is also localised to the cilium; deletion of the C-terminal palmitoylation site of CK1γ prevents its ciliary accumulation and renders it unable to associate with SMO [115]. By controlling entry and exit of proteins, the primary cilium is able to regulate the pathway by compartmentally restricting pathway components. Furthermore, as receptors and activators of the pathway are in continuous flux, the pathway can be rapidly activated or repressed in response to ligand availability.

In the absence of ligand, signalling is repressed by PTC, which is localised within the ciliary membrane. Upon signal activation, PTC moves out of the cilium and is replaced by SMO [170,171] (Figure 2), the accumulation of which is mediated by β–arrestin and the IFT family member KIF3A [172]. Accumulation of SMO within the cilium is, however, not sufficient for pathway activation; SMO must be activated (as discussed previously) in order for it to promote downstream signalling [173]. Hedgehog signalling can therefore be regulated by either preventing accumulation of SMO within the cilium, or by inhibiting its activation, and this two-step process is thought to provide a way of preventing aberrant activation of the pathway [173]. Cilial entry of SMO also results in the removal of GPR161 from the cilium in a β–arrestin mediated manner [174]. Gpcr161 is an orphaned G-protein-coupled receptor which acts to negatively regulate the hedgehog pathway by modulating cAMP levels, so promoting GLI processing into its repressor form by PKA [175] (Figure 2). Once SMO has entered the cilial compartment, it interacts with EVC and EVC2. These proteins are restricted to a specific region close to the basal body, and loss of this compartmental restriction leads to inhibition of the hedgehog pathway [176,177]. EVC and EVC2 act upstream of SUFU to promote GLI activation, although the precise mechanism by which this occurs is yet to be fully elucidated.

The activation and accumulation of SMO leads to increased levels of the GLI proteins within the cilium, where they localise to the distal tip [162,163,178]. Prior to translocation into the cilium, the GLI proteins form a complex with SUFU, which is co-translocated following signal activation. At the ciliary tip, this complex dissociates, allowing the GLI transcription factors to translocate to the nucleus [146].

Correct localisation of the GLI proteins within cilia is mediated by the Cos2 orthologue KIF7. The role of KIF7 appears to be to regulate cilial growth by binding to the plus end of growing microtubules, promoting catastrophe [163]. This microtubule regulation generates the compartment at the ciliary tip, allowing components of the hedgehog pathway to be appropriately localised and processed [179]. The trafficking of GLI by IFT proteins in and out of the cilial compartment is necessary to generate both activator and repressor forms of these transcription factors [180], and is therefore indispensable for the correct regulation of vertebrate hedgehog signalling.

## 10. Perspectives

This review has highlighted nodes of regulation that can enhance or inhibit output of the hedgehog signalling pathway. However, presenting one pathway in isolation does not give a true picture of cell processes in the context of a developing embryo or an adult tissue. During cellular response, input from other environmental cues and signals must be integrated, some of which can enhance the effect of Shh—such as the effect of Notch, which up-regulates SMO activity [181,182]. In addition, other signals can change the way a cell responds to Shh, as seen in neural precursors that are specified as floorplate at the intersection of FGF and Shh signalling in the posterior ventral neural plate [183]. These other pathways also include multiple levels of regulation that will also impact Shh signalling. Taken together with the mechanisms described, there is evidence for a multi-layered, complex system regulating this important signalling pathway.
